# Evolution of Direct Costs in the First Years of Rheumatoid Arthritis: Impact of Early versus Late Biologic Initiation - An Economic Analysis Based on the ESPOIR Cohort

**DOI:** 10.1371/journal.pone.0097077

**Published:** 2014-05-08

**Authors:** Karine Chevreul, Georges Haour, Sandy Lucier, Stephanie Harvard, Marie-Laure Laroche, Xavier Mariette, Alain Saraux, Isabelle Durand-Zaleski, Francis Guillemin, Bruno Fautrel

**Affiliations:** 1 URC Eco Île-de-France (AP-HP), Hôtel Dieu, Paris, France; 2 Inserm, ECEVE, U1123, Paris, France; 3 Univ Paris Diderot, Sorbonne Paris Cité, ECEVE, UMRS 1123, Paris, France; 4 Pierre and Marie Curie University (UPMC) – Paris 6, UPMC GRC 08, Institut Pierre Louis d’Epidémiologie et Santé Publique, Paris, France; 5 Pharmacology and Toxicology Unit, Centre of Pharmacovigilance-Pharmacoepidemiology CHU, Limoges, France; 6 Limoges University, EA 6310 HAVAE, Faculty of Medicine, Limoges, France; 7 Paris XI University, Department of Rheumatology, Bicêtre University Hospital (AP-HP), Le Kremlin Bicêtre, France; 8 Brest University, Department of Rheumatology, La Cavale Blanche Hospital, Brest, France; 9 Lorraine University, EA 4360 APEMAC; INSERM CIC-EC CIE6, Nancy, France; 10 Department of Public Health, Henri Mondor-Albert Chenevilier Hospitals (AP-HP), Créteil, France; 11 AP-HP, Department of Rheumatology, La Pitié Salpêtrière University Hospital, Paris, France; Center for Rheumatic Diseases, India

## Abstract

**Objectives:**

To estimate annual direct costs of early RA by resource component in an inception cohort, with reference to four distinct treatment strategies: no disease modifying antirheumatic drugs (DMARDs), synthetic DMARDs only, biologic DMARDs in the first year (‘first-year biologic’, FYB), and biologic DMARDs from the second year after inclusion (‘later-year biologic’, LYB); to determine predictors of total and non-DMARD related costs.

**Methods:**

The ESPOIR cohort is a French multicentric, prospective study of 813 patients with early arthritis. Data assessing RA-related resource utilisation and disease characteristics were collected at baseline, biannually during the first two years and annually thereafter. Costs predictors were determined by generalised linear mixed analyses.

**Results:**

Over the 4-year follow-up, mean annual direct total costs per treatment strategy group were €3,612 for all patients and €998, €1,922, €14,791, €8,477 respectively for no DMARDs, synthetic DMARDs only, FYB and LYB users. The main predictors of higher costs were biologic use and higher Health Assessment Questionnaire (HAQ) scores at baseline. Being a biologic user led to a higher total cost (FYB Rate Ratio (RR) 7.22, [95% CI 5.59–9.31]; LYB RR 4.39, [95% CI 3.58–5.39]) compared to non-biologic users. Only LYB increased non-DMARD related costs compared to all other patients by 60%.

**Conclusions:**

FYB users incurred the highest levels of total costs, while their non-DMARD related costs remained similar to non-biologic users, possibly reflecting better RA control.

## Introduction

Rheumatoid arthritis (RA) is an autoimmune disease causing chronic inflammation of the joints, often resulting in severe functional disability. The prevalence of RA in most world regions is under 1% of the population, [1] with an incidence of 0.2‰ to 0.5‰ [2].

In France, the mean annual direct cost of RA was estimated (costs are expressed in €2007 by applying the evolution of the consumption of care and medical goods price index in France. [3]) at €4,437 per patient in 2000 [4] and €9,345 in 2005. [5] Direct costs increased following the introduction of highly effective biologic disease modifying anti-rheumatic drugs (DMARDs), available since 2000 at more than ten times the cost of synthetic DMARDs. [6] With the expanded use of biologics, drugs are beginning to replace in-patient care as the greatest source of direct costs, [7]–[9] and differences in access to biologics will likely become the key variable in RA cost of illness across countries. [7] Used in patients with severe disease, biologics are currently not recommended as a first-line therapy due to safety and cost concerns. [10] While model results have demonstrated that early biologic initiation results in lower costs and improved quality-adjusted life expectancy, [11], [12] real-life data are lacking on the mid- to long-term impact of biologics on the direct costs of RA, including the time of initiation of biologic therapy. Furthermore, cost of illness data from larger samples and among patients with early or mild disease is needed, as the majority of studies to-date have included only patients with established RA [13].

The *Etude et Suivi des Polyarthrites Indifférenciées Récentes* (ESPOIR) cohort is a multicentric, prospective study of French patients with early arthritis (EA). [14] Our objective was to estimate the annual direct costs of RA among patients in the ESPOIR cohort in their first four years of illness, to describe the distribution of costs by resource component and to identify factors associated with costs. In order to explore the economic and clinical impact of the time of biologic initiation, we sought to compare direct costs and disease activity between RA patients prescribed a biologic in their first year of disease treatment (‘first-year biologic’, FYB) and those prescribed a biologic later, after their first year of treatment (‘later-year biologic’, LYB).

## Patients and Methods

### Patients

The ESPOIR cohort recruited 813 patients in France between 2002 and 2005. [14] Patients were aged 18–70 years, had a rheumatologist’s diagnosis of probable or certain RA or EA potentially becoming RA, with at least two swollen joints and symptom onset – first persistent arthritis according to the patient - between six months and six weeks before inclusion. History of DMARD use was limited to within two weeks before inclusion, while corticoid use was limited to a two-week prescription duration at a maximum mean dose of 20 mg/day of prednisone (four weeks for intra-articular injections). Due to the mode of recruitment, the cohort may be considered representative of the EA patient population in France and other similar countries. Patients were followed at one of 14 hospital centres every six months for a period of two years and every year thereafter. At each study visit after baseline, data were collected regarding health resource use since the previous visit, while data on patient sociodemographics, disease severity (health assessment questionnaire (HAQ) and Disease Activity Score on 28 joints (DAS-28) scores) and RA management were collected at every visit. The ethics committee of Montpellier approved the ESPOIR research programme in July 2002. All the patients signed an informed consent form before inclusion.

### Costing Method: Definition and Sources of Costs

Our analysis included only direct RA costs from the French health system perspective, all three payers combined (i.e., statutory health insurance (SHI), complementary health insurance, and patients), divided between the cost of RA-specific drugs (either synthetic or biologic DMARDs) and costs of other healthcare resources. The cost of DMARDs was defined as the cost of the drugs themselves plus costs related to administration (e.g., day hospitalisations for intravenous biologics). Other healthcare costs included consultations with physicians and other health professionals, symptomatic treatments using non-DMARD drugs, imaging (X-rays, computerised tomography (CT scans), magnetic resonance imaging (MRI), gastric endoscopies, colonoscopies), transportation, hospitalisations (except for biologic administration -Infliximab, Rituximab, Abatacept – administered exclusively in a hospital setting) and clinical workups including blood tests. Because costs associated with the DRG coding version used were available only until 2007, costs were expressed using €2007 [3].

For both specialists and general practitioners, an average consultation fee was estimated using 2007 SHI tariffs and average extra billing. [15], [16] Mean psychologist fees were derived from a survey. [17] Visits to physiotherapists were valued as the average tariff for patient education in inflammatory arthritis. [18] Nurse visits were valued as the tariff for 30 minutes of care by a skilled nurse plus a fixed average travel allowance [18].

Clinical workups were assigned their 2007 values based on expert opinion for inclusion, monthly and annual blood tests. [18] Transportation (ambulance and patient transport service) was valued by calculating an average cost per km and using a national average distance of 20 km to visit a rheumatologist [19].

Drugs were grouped according to their international nonproprietary names (INN), and their costs were calculated by estimating mean tariffs per milligram or per dose, weighted for the volumes sold in France in 2007 based on claims data. [18] The results were then multiplied by dosage and by prescription duration in days. Costs were estimated in each of the four treatment strategies, with patients divided into four mutually exclusive groups, the first two dividing non-biologic users into two groups and the last two dividing biologic users into two groups as well: ‘no biologic use’, synthetic DMARDs only, ‘first-year biologic’ (FYB) and ‘later-year biologic’ (LYB).

Hospitalisation costs were estimated according to diagnosis-related groups (DRG) using 2007 [20], [21] cost estimations and were adjusted by the number of days. Costs were calculated for each six-month (or one-year) period and then summed in order to obtain the total cost over four years and the mean annual per-patient cost.

### Statistical Analyses

Patient characteristics were described by the mean (SD) or number (%) and costs by the mean ± SD (median). Group comparisons were made using the Student t-test, the Wilcoxon Mann Whitney-test or the χ^2^-test.

The clinical impact of the time of biologic initiation among biologic users was assessed by comparing the proportion of patients with low disease activity (DAS-28≤3.2), the proportion of patients needing assistance from other personnel, the proportion of patients with RA caused disability, mean HAQ and mean EuroQol Five Dimensional Questionnaire (EQ-5D-3L) throughout the study period in groups of FYB and LYB users matched on baseline characteristics using a logistic regression propensity score the variables of which were selected using the stepwise selection method.

A regression analysis was conducted to identify determinants of total direct costs in the first four years of illness for patients who had attended all follow-up visits. To assess the timeliness of biologic initiation, another regression analysis investigated determinants of other (non-DMARD) healthcare costs that reflect the patients’ healthcare needs and level of disease activity. Because of the skewed distributions of both primary outcomes and the hierarchical structure of the data by center, we used generalised linear mixed models, which have been recommended for this type of cost data analysis. [22] Specifically, the fitted models were random intercept gamma regression models with log links. Coefficients were exponentiated to express effects as rate ratio (RR) estimates. For each model, the proportion of variance explained was calculated as the total variance from the fully unconditional model minus the total variance from the conditional model, divided by the total variance from the fully unconditional model. [23] The intraclass correlation coefficient (ICC), which is the proportion of the total variance that is accounted for by the center level, for each model was calculated as well. Variables were selected using the backward elimination method.

Variables used to investigate potential cost factors and to calculate propensity scores included patient sociodemographics, disease severity and RA management characteristics. Sociodemographic variables included age, sex, marital status, income and health insurance coverage. Disease severity indicators included hospitalisation at baseline or in the preceding six months (yes/no), functional ability at baseline expressed in four mutually exclusive groups (HAQ≤0.5; 0.5<HAQ≤1; 1<HAQ≤2; HAQ>2), [24] variation in HAQ in the first six months, presence of anti-cyclic citrullinated peptide (ACPA), presence of IgM rheumatoid factor at baseline and intensity of pain on a scale of 1 to 6. Effects of the DAS-28 score on costs were not investigated because it was highly correlated with the HAQ score (Pearson correlation coefficient = 0.60, p<0.0001), a fundamental outcome measure in RA. [24] Moreover, DAS-28 constitutes one of the main criteria for determining DMARD treatment strategies and thus would be a confounding factor. [25] RA management characteristics included physician certainty of RA diagnosis on a 0–100 visual analogue scale and treatment strategy.

Dropouts and missed study visits may be linked to patients’ disease severity, and thus missing data may lead to bias in cost analyses. To address this issue and assess the robustness of the models, sensitivity analyses were conducted using a Markov chain Monte Carlo (MCMC) multiple imputation (24 cycles) method based on a multivariate normal distribution. Patients who missed all study visits and those who died during the study period remained excluded. Imputations concerned log-transformed costs per period (12.3% of missing data) as well as the variables FYB (4.5%) and LYB (22.5%), which were imputed without rounding. [26] The following variables were used in the imputation model: all variables tested for the complete case analyses as well as dummy variables to account for the multicentric structure of the data [27] and supplementary auxiliary variables correlated with the variables to be imputed as well as with their missingness. [28] Data were assumed to be missing at random. Imputed costs per period were back transformed and then summed. Regression analyses were performed on each imputed data set, and results were combined using Rubin’s rules [29].

Statistical analyses were performed using SAS 9.3 (SAS Institute, Cary, North Carolina).

## Results

### Study Population

Two-thirds (n = 548) of the 813 patients in the ESPOIR cohort attended all follow-up visits and were included in the baseline analysis (Table 1). The majority (71.0%) were treated with synthetic DMARDs only, while 9.3% received no DMARDs, 7.7% received a FYB and 12.0% received a LYB. LYB received biologics with a median duration of 2 years after inclusion (mean duration: 2.29 years, SD: 0.96).

**Table 1:** Baseline patient characteristics.

			Study population stratified on treatment strategy			
	Whole pop.	Study pop.	No DMARDs	Synthetic DMARDs only	FYB	LYB
	(n = 813)	(n = 548)	(n = 51)	(n = 389)	(n = 42)	(n = 66)
**Age in years**	47.6 (12.6)	49.2 (11.6)	50.6 (11.1)	49.9 (11.5)	46.7 (12.2)	45.8 (11.9)
**Female**	624 (76.8%)	422 (77.0%)	36 (70.6%)	305 (78.4%)	33 (78.6%)	48 (72.7%)
**Married**	503 (61.9%)	358 (65.3%)	36 (70.6%)	258 (66.3%)	24 (57.1%)	40 (60.6%)
**High school diploma**	255 (31.4%)	171 (31. 2%)	19 (37.3%)	120 (30.9%)	11 (26.2%)	21 (31.8%)
**Population of place of residence**						
<5.000	274 (33.7%)	199 (36.3%)	24 (47.1%)	137 (35.2%)	12 (28.6%)	26 (39.4%)
>5.000 and <20.000	152 (18.7%)	100 (18.3%)	7 (13.7%)	76 (19.5%)	5 (11.9%)	12 (18.2%)
>20.000	366 (27.8%)	249 (45.4%)	20 (39.2%)	176 (45.2%)	25 (59.5%)	28 (42.4%)
**Monthly household income (€)**						
not reported	35 (4.2%)	20 (3.7%)	2 (3.9%)	11 (2.8%)	2 (4.8%)	5 (7.6%)
<1.220	154 (19.0%)	83 (15.2%)	9 (17.7%)	51 (13.1%)	5 (11.9%)	18 (27.3%)
>1.220 and <1.830	171 (21.1%)	114 (20.8%)	11 (21.6%)	83 (21.3%)	9 (21.4%)	11 (16.7%)
>1.830 and <2.440	156 (19.2%)	117 (21.4%)	12 (23.5%)	84 (21.6%)	9 (21.4%)	12 (18.2%)
>2.440 and <2.745	69 (8.5%)	53 (9.7%)	3 (5.9%)	43 (11.1%)	3 (7.1%)	4 (6.1%)
>2.745	228 (28.1%)	161 (29.4%)	14 (27.5%)	117 (30.1%)	14 (33.3%)	16 (24.2%)
**Full health coverage**	741 (91.1%)	510 (93. 1%)	46 (90.2%)	364 (93.6%)	38 (90.5%)	62 (93.9%)
**Symptom duration (days)**	214 (253)	102.1 (50.9)	103.2 (44.7)	98.5 (49.9)	109.8 (68.7)	117.8 (44.8)
**Swollen joint count**	7.19 (5.4)	7.5 (5.4)	5.8 (5.5)	7.5 (5.4)	9.8 (5.8)	7.8 (4.8)
**Tender joint count**	8.43 (7.0)	8.5 (7.0)	5.8 (5.9)	8.3 (7.1)	11.7 (6.8)	9.9 (6.7)
**ESR (mm/1st hour)**	29.43 (24.6)	29.3 (24.6)	19.9 (23.1)	29.0 (24.2)	40.6 (28.2)	30.9 (22.6)
**CRP (mg/l)**	22.15 (33.6)	21.6 (32.0)	14.5 (23.6)	21.2 (32.3)	28.2 (32.4)	25.2 (35.2)
**IgM RF positive**	372 (45.7%)	276 (50.3%)	8 (15.7%)	190 (48.8%)	31 (73.8%)	47 (71.2%)
**ACPA positive**	315 (39.0%)	250 (45.6%)	4 (7.8%)	168 (43.2%)	31 (73.8%)	47 (71.2%)
**Typical damage on X-ray**	110 (13.6%)	84 (15.3%)	2 (3.9%)	58 (14.9%)	11 (26.2%)	13 (19.7%)
**DAS 28**	5.11 (1.30)	5.12 (1.30)	4.27 (1.45)	5.08 (1.23)	5.95 (1.16)	5.49 (1.27)
**HAQ**	0.97 (0.68)	0.97 (0.68)	0.74 (0.65)	0.94 (0.67)	1.37 (0.76)	1.10 (0.65)
**Satisfaction of ACR criteria (1987)**	600 (73.8%)	417 (76.1%)	23 (45.1%)	305 (78.4%)	38 (90.5%)	51 (77.3%)
**Satisfaction of ACR criteria (2010)**	641/811 (79.0%)	456 (83.2)	25 (49.0%)	330 (84.8%)	42 (100%)	59 (89.4%)
**Physician diagnostic certainty (0–100 VAS)**	67.9 (24.5)	71.8 (23.8)	52.5 (23.9)	71.8 (23.6)	81.4 (16.8)	80.7 (19.2)
**1st rheumatologist visit after RA onset** 1	74.9 (76.6)	76.9 (83.2)	63.1 (47.2)	75.9 (79.6)	110.8 (151.2)	70.7 (55.8)
**DMARD initiation at 6-month visit**	599/757 (79.1%)	452 (82.5%)	0 (0%)	353 (90.8%)	42 (100%)	57 (86.4%)
**Hospitalization before baseline**	193 (23.9%)	133 (24.4%)	11 (21.6%)	90 (23.1%)	15 (35.7%)	17 (25.8%)

Values are mean values (SD) or number of patients (%).

1 Eular Guideline: 1st rheumatologist visit after RA onset <45 days; ESR = Erythrocyte sedimentation rate, CRP = C-Reactive Protein, DAS-28 = Disease Activity Score 28, HAQ = Health Assessment Questionnaire, ACR = American College of Rheumatology, VAS = Visual analogue scale, EULAR = European League Against Rheumatism, DMARD = Disease-modifying antirheumatic drug, ACPA = anti-citrullinated protein antibody.

Among the 265 excluded patients, baseline HAQ and DAS-28 scores were not different from the study population’s (p = 0.52 and p = 0.57, respectively), nor was sex distribution (p = 0.80). However, excluded patients were slightly younger (45 vs. 49 years, p = 0.002), fewer were positive for ACPA (24.5% vs. 45.6%, p<0.0001) and IgM rheumatoid factor (IgM RF) (36.2% vs. 50.4%, p = 0.0001) and fewer met the 1987 American College of Rheumatology (ACR) RA criteria (61.1% vs. 76.1%, p<0.0001).

The study population for the regression sensitivity analyses (n = 777) included the 813 cohort patients, less the seven who died during the study period and the 29 patients who attended no study visits following inclusion.

### Impact of Time of Initiation of Biologics on Disease Activity

**Figure 1:**
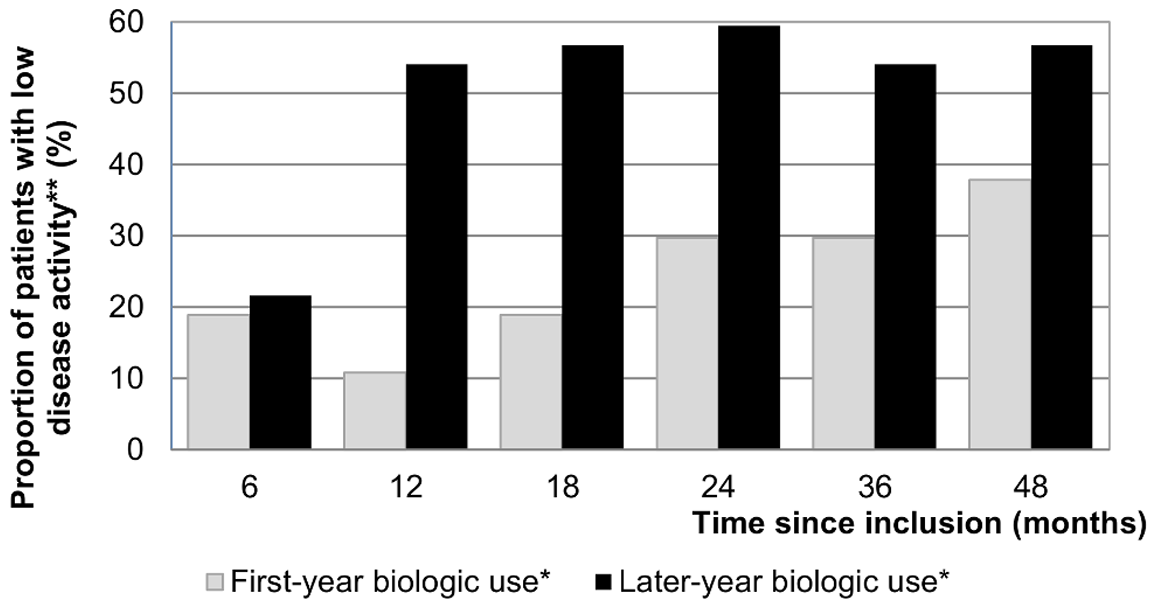
Disease activity per period among matched groups of FYB and LYB users. * 37 FYB users and 37 LYB users were matched for clinical and sociodemographic baseline characteristics using a logistic regression propensity score. ** Low disease activity is defined as DAS-28≤3.2.

A comparison of groups of FYB and LYB users matched on baseline characteristics (baseline DAS-28 and HAQ scores not significantly different among two groups of equal sizes (n = 37) with p = 0.93 and p = 0.68 respectively after matching) shows that over the entire study period, FYB users compared to LYB users display lower proportions of patients with low disease activity (DAS-28≤3.2) (see Figure 1), lower proportions of patients needing assistance for daily activities, lower proportions of patients with RA caused disabilities, lower mean HAQ scores and higher mean EQ-5D-5L scores (see Table 2).

**Table 2:** Indicators comparing matched groups of FYB and LYB users per period.

	Time since inclusion (months)						
	0	6	12	18	24	36	48
**Assistance needed in the last 6 months − (n (%))**							
First-year biologic (n = 37)*	2 (5.41%)	4 (10.81%)	4 (10.81%)	3 (8.11%)	3 (8.11%)	5 (13.51%)	4 (10.81%)
Later-year biologic (n = 37)*	6 (16.22%)	5 (13.51%)	4 (10.81%)	3 (8.11%)	6 (16.22%)	7 (18.92%)	11 (29.73%)
**RA caused disability - (n (%))**							
First-year biologic	0 (0%)	1 (2.7%)	1 (2.7%)	2 (5.41%)	4 (10.81%)	7 (18.92%)	8 (21.62%)
Later-year biologic	0 (0%)	1 (2.7%)	2 (5.41%)	3 (8.11%)	4 (10.81%)	9 (24.32%)	11 (29.73%)
**HAQ - (mean ± SD)**							
First-year biologic	1.29±0.77	0.92±0.65	0.67±0.59	0.66±0.34	0.67±0.61	0.71±0.65	0.76±0.66
Later-year biologic	1.36±0.64	0.95±0.57	1.03±0.63	0.68±0.36	0.95±0.65	0.91±0.54	0.84±0.55
**EQ-5D-5L utility - (mean ± SD)**							
First-year biologic	0.31±0.35	0.43±0.36	0.54±0.36	0.53±0.31	0.54±0.34	0.57±0.3	0.49±0.37
Later-year biologic	0.23±0.33	0.38±0.29	0.39±0.3	0.39±0.33	0.42±0.34	0.44±0.31	0.48±0.34

*37 FYB users and 37 LYB users were matched for clinical and sociodemographic baseline characteristics using a logistic regression propensity score.

The variables retained in the propensity score model were: satisfaction of ACR criteria, HAQ level and home ownership status.

### Descriptive Cost Analysis

**Figure 2:**
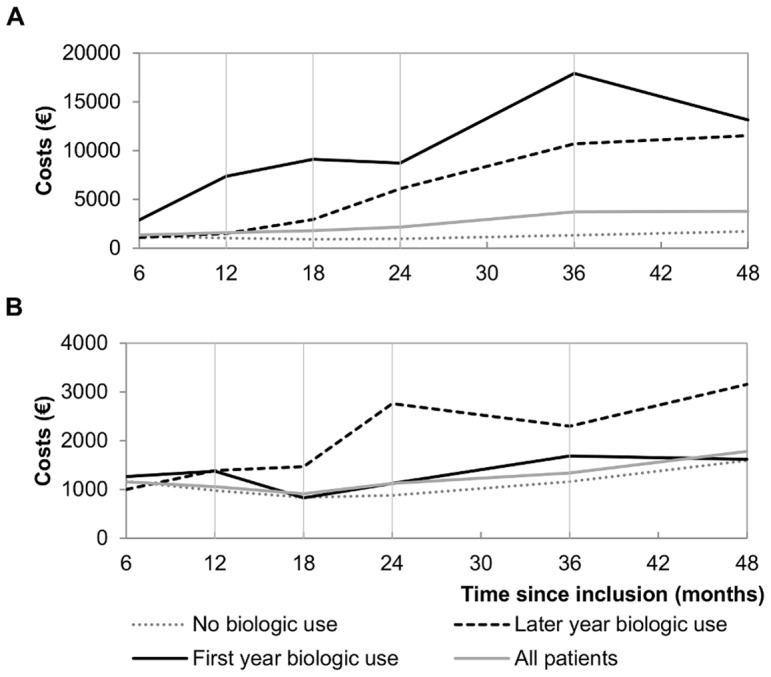
Mean costs per period according to treatment strategy. (A) Mean total costs according to treatment strategy; (B) Mean other health resource use costs according to treatment strategy.

The breakdown of costs by resource component is shown in Table 3. The mean annual total cost per patient over the four-year period was €3,612, with DMARD costs accounting for nearly half that amount. It can be seen in Figure 2 (A) that total costs increased year by year over the study period in all treatment strategy groups except for FYB users whose mean annual total costs decreased between year three and year four. Among other costs, hospitalisation was the largest component, constituting one-third of total costs with wide variation among groups. Hospitalisation costs for LYB users were more than three times higher than those of patients not treated with DMARDs.

**Table 3:** Annual costs over the 4-year follow-up by resource component.

				Stratification on RA therapeutic strategy							
	Study population (n = 548)			No DMARD (n = 51)		Synthetic DMARD only (n = 389)		First-year biologic (n = 42)		Later-year biologic (n = 66)	
	Mean ± SD (median)	%		Mean ± SD (median)	%	Mean ± SD (median)	%	Mean ± SD (median)	%	Mean ± SD (median)	%
**DMARDs/Biologics**	1,767±4,141 (95)	49.0		0±0	0.0	180±231 (84)	9.4	12,816±5,760 (11,294)	86.6	5,462±3,582 (5,381)	64.4
**Other healthcare use**	1,844±3,251 (974)	51.0		998±1,147 (622)	100	1,742±3,478 (903)	90.6	1,974±1,639 (1,696)	13.4	3,016±3,490 (1,640)	35.6
All provider visits	318±406 (233)	8.8		174±147 (128)	17.4	308±432 (228)	16.0	382±336 (267)	2.6	446±382 (363)	5.2
Physicians^1^	200±14 (181)	5.5		127±100 (103)	12.8	195±107 (174)	10.1	224±118 (201)	1.5	269±125 (241)	3.2
Other health professionals^2^	118±354 (28)	3.2		46±94 (3)	4.6	113±385 (17)	5.9	158±289 (39)	1.1	177±324 (80)	2.1
Laboratory testing^3^	231±118 (218)	6.4		84±57 (72)	8.4	233±106 (223)	12.1	269±122 (243)	1.8	303±126 (288)	3.6
Imaging & other devices^4^	49±47 (35)	1.3		38±39 (30)	3.8	48±45 (34)	2.5	51±39 (36)	0.3	63±65 (42)	0.7
Transportation^5^	10±39 (0)	0.3		2±8 (0)	0.2	9±38 (0)	0.5	17±56 (0)	0.1	21±43 (0)	0.2
Inpatient care^6^	1,130±2,931 (176)	31.3		631±910 (95)	63.2	1,038±3,130 (95)	54.0	1,141±1,514 (552)	7.7	2,057±3,528 (796)	24.3
Non-DMARD medication	106±89 (88)	2.9		71±80 (37)	7.0	106±87 (89)	5.5	116±103 (105)	0.8	125±90 (103)	1.5
**Total annual costs**	3,612±5,407 (1423)	100		998±1,147 (62)	100	1,922±3,486 (1,146)	100	14,791±6,033 (13,726)	100	8,477±4,741 (8,121)	100

1 Physician visit costs used were the following: 28.27€ for rheumatologists, 28.61€ for internists, 22.13€ for general practitioners and 30.56€ for orthopaedist surgeons.

2 Health professional consultation costs used were the following: 11.30 € for nurses, 16.32 € for physiotherapists, 40.82 € for psychologists and 14.90 € for occupational therapists.

3 Lab test costs used were the following: 173.80 € for inclusion biologic investigations, 28.86 € for monthly biologic tests and 42.63 € for annual biologic tests.

4 Imaging and other devices costs included the following: 19.95 to 39.95 € for X-ray depending on the number of X-rays (19.95 € for the 1st X-ray, 39.95 € for the 2nd, 21.28 € for the 3rd, 20.00 € for the 4th), 69.00 € for MRI, 25.27 € for CT-scanner, 96.00 € for gastric endoscopy and 204.18 € for colonoscopy.

5 Transportation costs were calculated on the basis of a reimbursement fee of 1.17 € per km.

6 Inpatient costs were based on DRG tariffs.

DMARD = Disease-modifying antirheumatic drugs.

For patients receiving biologics, DMARDs constituted the highest share of total costs. For FYB patients the mean annual cost per patient was more than five times higher than that of the rest of the population, nearly twice that of LYB users and nearly eight times that of patients receiving only synthetic DMARDs.

Other healthcare costs were one-third lower among FYB patients compared to LYB patients, representing an average annual offset of €1,042. Healthcare costs of FYB patients remained comparable to patients who received no biologic treatment (Figure 2 (B)), although FYB patients were likely to have a more active disease at inclusion (mean DAS-28∶5.95 vs. 4.99, p<0.0001 and mean HAQ: 1.37 vs. 0.91, p<0.0001; see Table 1). This contrasted with LYB patient costs, which remained higher than those in all other groups and increased regularly over the four-year period with a peak increase 24 months after inclusion.

Grouping LYB patients by year of biologic initiation revealed that their total mean annual costs decreased with later initiation of biologics (total costs in order of year of biologic initiation: €10,661 (n = 32, 2^nd^ year after inclusion), €7,592 (n = 16, 3^rd^ year after inclusion) and €5,384 (n = 18, 4^th^ year after inclusion) respectively), while their other mean annual costs remained comparable (healthcare costs by year of biologic initiation: €3,032, €2,941 and €3,053 respectively). Inferential statistics were not produced due to the small size of these groups.

### Factors Associated with Costs

The results of the multivariate regression analyses of total costs and other healthcare costs are shown in Tables 4 and 5. The reported RR describe the variations in costs expressed as a multiplicative factor for patients presenting the associated characteristic compared to those who did not, all other things being equal.

**Table 4:** Multivariate analysis of the effects of demographic and clinical variables and treatment strategy on total costs over 4 years.

	Complete case analysis(n = 548)		MCMC imputation model(n = 777)	
Variables	Rate Ratio (95% CI)	P-value	Rate Ratio (95% CI)	P-value
**First year biologic use**	7.22 (5.59–9.31)	<0.0001	7.74 (5.70–9.78)	<0.0001
**Later year biologic use**	4.39 (3.58–5.39)	<0.0001	4.54 (3.38–5.70)	<0.0001
**HAQ score at baseline: >2**	2.25 (1.76–2.88)	<0.0001	2.10 (1.52–2.67)	0.0002
**HAQ score at baseline: 1 to ≤2**	1.45 (1.23–1.69)	<0.0001	1.50 (1.22–1.78)	0.0006
**Variation in HAQ score (per 0.25-point increase) between** **baseline and 6 month visit**	1.07 (1.03–1.10)	<0.0001	1.06 (1.03–1.10)	0.0007
**Doctor's evaluation of RA diagnostic <50% at baseline**	0.73 (0.62–0.86)	0.0002	0.78 (0.65–0.91)	0.0014
**Hospitalisation at baseline**	1.32 (1.13–1.54)	0.0005	1.29 (1.07–1.50)	0.0083
**Lives with a partner at baseline**	0.67 (0.57–0.79)	<0.0001	0.69 (0.58–0.81)	<0.0001
**Age (per 10-year increase) at baseline**	–	–	1.11 (1.05–1.18)	0.0009
**Full health coverage**	–	–	0.67 (0.48–0.87)	0.0014
**Family income>€1220**	–	–	1.28 (1.03–1.52)	0.0264
**Proportion of variance explained by model**	53%		46% (44%–48%)^1^	
**ICC**	4.15%		5.97% (4.05%–8.53%)^1^	

The reported Rate Ratios describe the variations in costs expressed as a multiplicative factor for patients presenting the associated characteristic compared to those who did not, all other things being equal.

MCMC = Markov Chain Monte Carlo; CI = Confidence Intervalle; HAQ = Health Assessment Questionnaire; RA = Rheumatoid Arthritis; ICC = Intraclass Correlation Coefficient.

1 Median and range for all 24 imputed datasets.

**Table 5:** Multivariate analysis of the effects of demographic and clinical variables and treatment strategy on other health resource use costs over 4 years.

	Complete case analysis(n = 548)		MCMC imputation model(n = 777)	
Variables	Rate Ratio (95% CI)	P-value	Rate Ratio (95% CI)	P-value
**Later year biologic use**	1.64 (1.32–2.05)	<0.0001	1.73 (1.28–2.18)	0.0016
**HAQ score at baseline: >2**	2.43 (1.87–3.17)	<0.0001	2.66 (1.87–3.45)	<0.0001
**HAQ score at baseline: 1 to ≤2**	1.48 (1.24–1.76)	<0.0001	1.51 (1.22–1.81)	0.0007
**Variation in HAQ score (per 0.25-point increase) between** **baseline and 6 month visit**	1.06 (1.03–1.10)	0.000	1.08 (1.04–1.11)	<0.0001
**Doctor's evaluation of RA diagnostic <50% at baseline**	0.76 (0.64–0.92)	0.004	0.82 (0.67–0.96)	0.0114
**Hospitalisation at baseline**	1.38 (1.16–1.64)	0.000	1.29 (1.06–1.51)	0.0113
**Lives with a partner at baseline**	0.65 (0.55–0.77)	<0.0001	0.72 (0.60–0.84)	<0.0001
**Age (per 10-year increase) at baseline**	–	–	1.10 (1.03–1.17)	0.0033
**Proportion of variance explained by model**	22%		17% (16%–19%)^1^	
**ICC**	4.85%		5.85% (4.21%–8.02%)^1^	

The reported Rate Ratios describe the variations in costs expressed as a multiplicative factor for patients presenting the associated characteristic compared to those who did not, all other things being equal.

MCMC = Markov Chain Monte Carlo; CI = Confidence Intervalle; HAQ = Health Assessment Questionnaire; RA = Rheumatoid Arthritis; ICC = Intraclass Correlation Coefficient.

1 Median and range for all 24 imputed datasets**.**

The total and other healthcare costs models were similar in terms of significance and magnitude of effects. Higher HAQ scores at baseline, increase in HAQ score between baseline and the six-month study visit and hospitalisations at baseline or in the six months prior to inclusion were associated with higher costs, whereas living with a partner and having a doctor’s certainty of RA diagnostic below 50% had the opposite effect. The strongest effects on costs were found for the use of biologic treatments and HAQ.

While the biologic treatment strategy induces the highest increase in total costs, with FYB showing a greater impact than LYB, only LYB shows a significant relation with other healthcare costs: plus 60% compared to all other patients.

Sensitivity analyses showed similar results but with additional significant effects. Higher age at baseline increased both types of cost, while high family income and full health coverage were only related to total costs with respectively increasing and decreasing effects.

The total costs model explained more than 50% of observed variations, while only 22% were explained by the other healthcare cost model. Furthermore, those variations were primarily driven by differences between patients rather than between medical centres’ practices, with intraclass correlation coefficients lower than 5% when adjusted for patient-level variables.

## Discussion

Our study reported the costs of a large sample of EA patients, more than 80% of whom satisfied the RA classification criteria. [30] With 71% of patients using only synthetic DMARDs and 19.7% using biologics, mean annual total costs in the cohort were €3,612 while mean other healthcare costs were €1,844.

High costs of RA in several countries have been described from both the societal and public payer perspectives, [7], [13] with the latter most often estimating only direct costs of RA treatment. A recent overview in western countries estimated the average annual direct costs in €2007 at €4,208, [13] while another reported substantially higher costs in western European countries (€15,135) than in central or eastern European countries, (€3,532). [7] The ESPOIR cohort’s direct costs were lower than those previously reported in the literature. [13], [31], [32] This could be partially explained by the fact that the present study explores the early stages of the disease, for which surgery and disability care are limited. Moreover, while previous studies found hospitalisations to be the largest cost share, [13], [33] we found that it is currently attributable to RA-specific drug costs, particularly biologic therapies, which represent almost half of the direct four-year costs. The economic impact is significant for the French statutory health insurance, which bears almost the full cost of these drugs.

Our analysis revealed wide variations in direct medical costs among patients in the first four years of RA treatment depending on the treatment strategy. As expected, total costs over four years were over 70% higher for patients with FYB use compared to patients with LYB use. This difference was due to the increased cost of biologic DMARDs, as other costs were in fact lower among FYB patients compared to LYB users over the period. As shown in Figure 1 (B), FYB users’ other healthcare costs remained at a lower level throughout the four-year study period than those of LYB users, indicating greater diminishment in disease progression among FYB users, which is supported by a higher proportion of patients with low disease activity (DAS-28≤3.2) (see Figure 2), lower rates of needed assistance and RA caused disability, higher HAQ levels and lower EQ-5D-3L utilities (see Table 2) among FYB users’ after matching on baseline characteristics. Furthermore, FYB patients’ other costs over four years did not differ significantly from those of patients who did not need biologic therapy, while they were more than 60% higher for LYB patients. This is consistent with previous findings according to which very early suppression of disease activity is likely to reduce patient costs [34].

As other authors have found [35], [36] another significant cost driver was disease severity, as revealed by high HAQ values. In addition, when physicians were more confident in the RA diagnosis, they were more likely to use a step-up strategy [37], [38] resulting in higher resource use.

We acknowledge some limitations regarding data quality, availability and follow-up and statistical analyses. Information related to blood tests and analgesic dosages was not recorded in a standardised manner, and thus we used standard packages defined by an expert. Transportation costs were also standardised, as covered distances were not reported. However, the impact of these limitations on the reported results should be small because they account for a minor proportion of total costs: blood tests (∼5%), analgesics (<2%) and transportation (<1%). There was a substantial degree of missing values, with 33% of patients missing at least one follow-up visit. However, sensitivity analyses found the models to be robust. In addition, one may question the impact memory bias [39] may have on the quality of the data collected retrospectively given the length of time between study visits. Moreover, the overall study period may have been too short to reveal the longer term impact of biologic therapies on costs. Finally, we used 2007 prices and not more recent data. However the mean annual total costs in the cohort for 2011, obtained by applying the evolution of the consumption of care and medical goods price index in France was estimated at €3,616 which is quite similar to our 2007 results [3].

In our statistical analyses, the multiple imputation method we used to address the problem of missing data in the sensitivity analyses may have resulted in bias. We undertook log transformations of costs per period prior to implementing the multiple imputation in order to approach normality and obtain exclusively positive imputed costs following back transformation. Although some experts recommend such transformations, [40] more recent research has drawn attention to the limitations of this method, including degradation of the relationships between variables in the imputation model resulting in bias, and suggested approaches based on flexible non-normal distributions [41].

Our analysis suggests that early use of biologics may reduce other healthcare costs by slowing the evolution of the disease. We do not know the extent to which the high direct costs incurred from early use of biologics costs may be balanced in part by other health care cost offsets due to the reduction in the need for surgery or other interventions beyond the fourth year of the disease. Moreover, recent price reductions for biologics could make them more cost-effective, [42] thereby decreasing resistance to early use that may improve patients’ lives.
